# T-cell based sieve analysis ties HLA A*02 to vaccine efficacy and IgA-C1 immune correlate in RV144 Thai trial

**DOI:** 10.1186/1742-4690-9-S2-O61

**Published:** 2012-09-13

**Authors:** T Hertz, A Gartland, H Janes, S Li, Y Fong, GD Tomaras, D Morris, D Geraghty, GH Kijak, PT Edlefsen, M Rolland, BB Larsen, S Tovanabutra, E Sanders-Buell, AC DeCamp, CA Magaret, H Ahmed, S Nariya, K Wong, H Zhao, W Deng, BS Maust, M Bose, S Howell, M Lazzaro, A Bates, E Lei, A Bradfield, G Ibitamuno, V Assawadarachai, RJ O'Connel, MS deSouza, S Nitayaphan, S Rerks-Ngarm, ML Robb, MJ McElrath, BF Haynes, NL Michael, PB Gilbert, JI Mullins, JH Kim

**Affiliations:** 1 Fred Hutchinson Cancer Research Center, Seattle, WA, USA; 2 Duke University School of Medicine, Durham, NC, USA; 3 US Military HIV Research Program, Silver Spring, MD, USA; 4 University of Washington, Seattle, WA, USA; 5 Royal Thai Army Component, AFRIMS, Bangkok, Thailand; 6 Duke University, School of Medicine, Durham, NC, USA

## Background

The RV144 trial showed an estimated 31% vaccine efficacy (VE) against HIV-1 infection. Two immunological correlates of risk were found in vaccine recipients: Envelope V1/V2 antibody titers and IgA binding to Envelope (Haynes et al., 2012).

## Methods

We conducted a CD8^+^ T-cell based sieve analysis of the V1/V2 region testing for differential escape from vaccine induced epitopes as predicted using computational methods. Breakthrough peptides that differed from predicted epitopes in the vaccine insert and had reduced HLA binding affinity were considered escapes.

## Results

Three of twelve epitopes in V1/V2 of the MN protein boost showed evidence of escape (start-positions 147, 163, 168 restricted by A*11 and A*02), with more escapes in vaccine recipients (p=0.018). We hypothesized that if escape was indicative of anamnestic responses, recipient’s HLA alleles should not modify VE. While VE was not different in A*11(+/-) subgroups (p=0.45), it was higher in A*02(+) versus A*02(-) participants (VE=54% vs. 3%, interaction p-value=0.05). Previous analysis showed that HIV variants matching the vaccine insert at site 169 (K169) (in V2, implicated in antibody binding) were preferentially excluded from infections in vaccine recipients (VE against K169=48%, p=0.0036). We found significant VE against K169 in only the A*02(+) subgroup (74%, p=0.001; p-value for difference A*02(+/-)=0.01). Reanalyzing the immune correlates within A*02(+/-) subgroups, we found a direct correlation between IgA-C1 titers and infection rate in A*02(-) participants (OR=2.07, p=0.0002), but not in the A*02(+) participants (OR=1.12, p=0.71; A*02(+/-) interaction p-value=0.05).

## Conclusion

Our exploratory analysis, driven by a T-cell based sieve effect in envelope V1/V2, revealed an association between an HLA class I allele and VE, suggesting that VE was restricted to A*02(+) participants and that IgA-C1 antibodies inhibited protective effects of other responses in A*02(-) participants. This highlights the importance of considering the effects of host genetics on VE in future HIV vaccine trials.

